# Structural Ensembles of Membrane-bound α-Synuclein Reveal the Molecular Determinants of Synaptic Vesicle Affinity

**DOI:** 10.1038/srep27125

**Published:** 2016-06-08

**Authors:** Giuliana Fusco, Alfonso De Simone, Paolo Arosio, Michele Vendruscolo, Gianluigi Veglia, Christopher M. Dobson

**Affiliations:** 1 Department of Chemistry, University of Cambridge, Lensfield Road, Cambridge CB2 1EW, UK; 2 Department of Life Sciences, Imperial College London, South Kensington, London SW7 2AZ, UK; 3 Department of Chemistry & Department of Biochemistry, Molecular Biology & Biophysics, University of Minnesota, 6-155 Jackson Hall 321 Church st. SE, Minneapolis, MN 55455, USA

## Abstract

A detailed characterisation of the molecular determinants of membrane binding by α-synuclein (αS), a 140-residue protein whose aggregation is associated with Parkinson’s disease, is of fundamental significance to clarify the manner in which the balance between functional and dysfunctional processes are regulated for this protein. Despite its biological relevance, the structural nature of the membrane-bound state αS remains elusive, in part because of the intrinsically dynamic nature of the protein and also because of the difficulties in studying this state in a physiologically relevant environment. In the present study we have used solid-state NMR and restrained MD simulations to refine structure and topology of the N-terminal region of αS bound to the surface of synaptic-like membranes. This region has fundamental importance in the binding mechanism of αS as it acts as to anchor the protein to lipid bilayers. The results enabled the identification of the key elements for the biological properties of αS in its membrane-bound state.

α-synuclein (αS) is a 140-residue protein that is associated with a range of highly debilitating neurodegenerative conditions, of which the most common is Parkinson’s disease (PD)^1^,^2^,^3^,^4^,^5^. A hallmark of PD is the formation of abnormal intracellular protein aggregates, known as Lewy bodies, which are largely composed of amyloid fibrils of αS^6^,^7^,^8^,^9^,^10^. In addition, point mutations in the αS gene and also gene duplications and triplications are associated with early onset familial forms of PD^11^,^12^. αS is abundant in red blood cells^13^ and also localises at the termini of neurons^14^. Although its function is still highly debated, it is believed to be involved in the regulation of the homeostasis of synaptic vesicles during neurotransmitter release^15^,^16^,^17^,^18^, and it is widely accepted that a crucial role is played by the interactions of αS with membranes in both physiological and pathological contexts^19^,^20^,^21^. *In vivo*, αS is partitioned between cytosolic and membrane-associated forms, under apparently strictly regulated equilibrium conditions^22^. Membrane interactions are also potent modulators of the propensity of αS to self-assemble into amyloid fibrils, with the kinetics of aggregation being enhanced by several orders of magnitude in some cases through the presence of lipid vesicles^23^. Understanding the structural and dynamical nature of the membrane-bound state of αS is therefore a major priority in order to elucidate the balance between functional and dysfunctional roles of this protein^15^,^21^. The dynamic nature of αS in both its cytosolic and membrane-bound states has, however, made such studies extremely challenging.

It is widely acknowledged that αS is an intrinsically disordered monomeric protein in its physiological cytosolic form^24^, and that membrane binding induces a transition such that specific regions of the protein adopt a significant level of α-helical structure^19^,^20^,^25^,^26^,^27^. This transition is favoured by a series of imperfect 11 residue repeats encoding for amphipathic class A2 lipid-binding α-helical segments in the region spanning the first 90 residues of the protein^3^. These sequence patterns enhance a promiscuous tendency to bind to a variety of lipid/detergent assemblies ranging from micelles and vesicles to cellular membranes^21^. Nuclear magnetic resonance (NMR) studies involving lipids that mimic key features of synaptic vesicles have revealed that αS binds to lipid bilayers in a multiplicity of distinct modes^20^,^26^,^28^. As a consequence of this variability, a range of structural architectures, including a pair of anti-parallel α-helices (residues 3–37 and 45–92)^29^ and a single extended α-helix (residues 9–89)^30^,^31^ have been identified in the structure of αS bound to lipids. To obtain a physiologically relevant structural characterisation of the membrane-bound state of αS in the context of synaptic vesicle regulation, therefore, it is important to select an appropriate system, and small unilamellar vesicles (SUVs) with carefully chosen mixtures of 1,2-dioleoyl-sn-glycero-3- phosphoethanolamine (DOPE), 1,2-dioleoyl-sn-glycero-3-phospho-L-serine (DOPS), and 1,2-dioleoyl-sn-glycero-3-phosphocholine (DOPC) lipids have been shown to be excellent models for synaptic vesicles. These SUVs, however, cannot be studied by the conventional high resolution techniques of structural biology such as X-ray crystallography, as they do not form suitable crystals, or solution-state NMR, as their slow tumbling rates prevent the detection of the majority of the signals from bound αS molecules. Indeed using solution-state techniques, NMR resonances can be only detected for residues in the disordered C-terminus, the region of the protein having the lowest membrane affinity, while resonances from the N-terminal region of the protein that interacts strongly with membranes are essentially undetectable^20^,^26^.

In order to overcome these problems, we recently employed a combination of solution and solid-state NMR (ssNMR) methods to characterise in detail the conformational properties of the elusive membrane-bound state of αS, and to probe the nature both of its ordered and disordered regions^25^. The results of these studies have identified three different regions of αS that play distinct and specific roles in its binding to synaptic-like SUVs. These regions include an N-terminal ‘membrane-anchor’ segment (the initial 25 residues, for which we have assigned by ssNMR the resonances of residues 6–25), which represents the primary binding region of αS and adopts a stable amphipathic helical state on the lipid bilayer surface, a central ‘sensor’ region of the protein (residue 26 to 98), which serves to modulate its overall affinity for the lipid vesicles, and a C-terminal region (residue 99 to 140), which remains largely unstructured and associated only weakly with the membrane surface^25^.

In the present study, we have employed chemical shifts (CS), measured using ssNMR, as restraints in molecular dynamics (MD) simulations to generate a structural ensemble of the membrane-anchor region of αS bound to synaptic-like vesicles. In particular, by using experimental ssNMR data determined for the full length protein bound to SUVs, we have characterised in atomic detail the ensemble of structures of the segment spanning residues 1 to 30 of αS. The N-terminal region of αS is fundamental for membrane binding^19^,^32^ as it acts as a membrane-anchor region that initiates the adhesion to the vesicle surface^25^. In order to identify the key structural and topological properties of this region within the full-length protein, we sampled the free energy surface (FES) of this membrane-bound state and subsequently combined this information with paramagnetic relaxation enhancement (PRE) measurements. The results reveal key molecular determinants of the interactions between αS and membrane surfaces that are vital for both physiological and pathological roles of this protein.

## Results

### Structural ensembles of the membrane-anchor region of αS

We characterised a structural ensemble of the first 30 residues of αS bound to the surface of SUVs composed of a mixture of acidic lipids consisting of DOPE, DOPS and DOPC lipids in a 5:3:2 molar ratio; such SUVs have been shown to be good mimics of synaptic vesicles in composition and size^20^,^25^,^26^. The structural refinement of this N-terminal region of the protein, denoted as αS_1-30_, has been obtained by using experimental chemical shifts from ssNMR as restraints in ensemble-averaged molecular dynamics simulations^33^,^34^, employing an established protocol based on four replicas^35^ that evolve simultaneously starting from random conformations. Samplings were carried out for 1 μs until convergence was observed for four parameters, namely the root mean square deviations (RMSDs) in the C^α^ Cartesian coordinates and in the backbone dihedral angles, the radius of gyration and the solvent accessible surface area (Fig. S1). The resulting structural ensemble showed good agreement between the experimental chemical shifts and those calculated using highly accurate predictor, SPARTA+^36^, which is based on a fundamentally different approach to that of the CamShift method^33^ used in our structural refinement procedure (Fig. S2). The back calculations indicate that the refined ensemble matches the experimental data with standard deviations that are within the statistical errors of SPARTA+ (Fig. S2), providing evidence of its validity.

**Figure 1:**
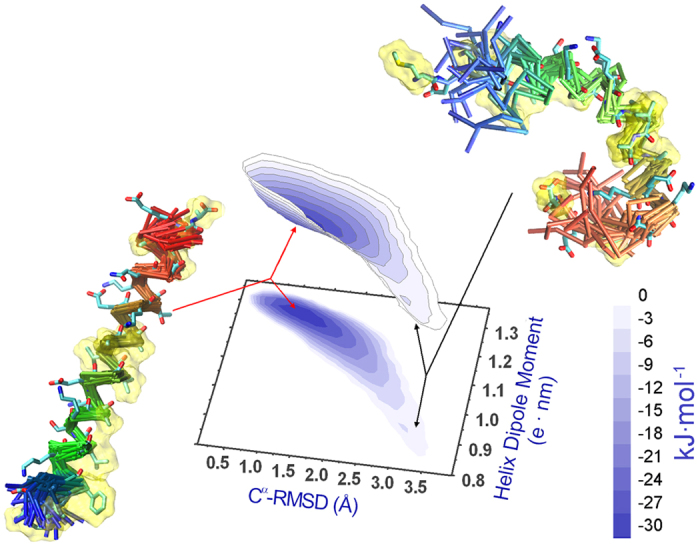
Free energy surface (FES) of the membrane-bound state of αS_1-30_. The ensemble was generated by means of molecular dynamics simulations restrained using ssNMR chemical shifts measured for the SUV bound state of full-length αS. The conformations are projected onto two reaction coordinates to define a two-dimensional free energy surface. The coordinates employed are the C^α^ root mean square deviation (RMSD) from an ideal helix and the dipole moment of αS_1-30_. Residues 1 to 5 and 26 to 30 have not been included in the calculations of the projection coordinates. The surfaces were generated by means of contour levels reproducing isosurfaces of free energy from 0.0 (white) to −30.0 (darkest blue) kJ/mol. Two representative structural bundles from the FES are shown.

We then projected the ensemble of αS_1-30_ onto two coordinates, the C^α^-RMSD deviation from an ideal helical conformation and the dipole moment of the structure, to obtain a free energy surface (FES) for this region of αS (Fig. 1). Overall the FES shows that the conformational heterogeneity of this region of αS is very significantly reduced when the protein is bound to lipid membranes, with this segment of the protein adopting a stable α-helical conformation characterised by a single free energy basin centred at a C^α^-RMSD of 1.0 Å from an ideal helix and a dipolar moment of 1.3 e.nm. In addition to the main basin, the FES also reveals a low-population conformation centred on an RMSD value greater than 3.0 Å from an ideal helix and a dipolar moment of *ca* 0.9 e.nm.

**Figure 2:**
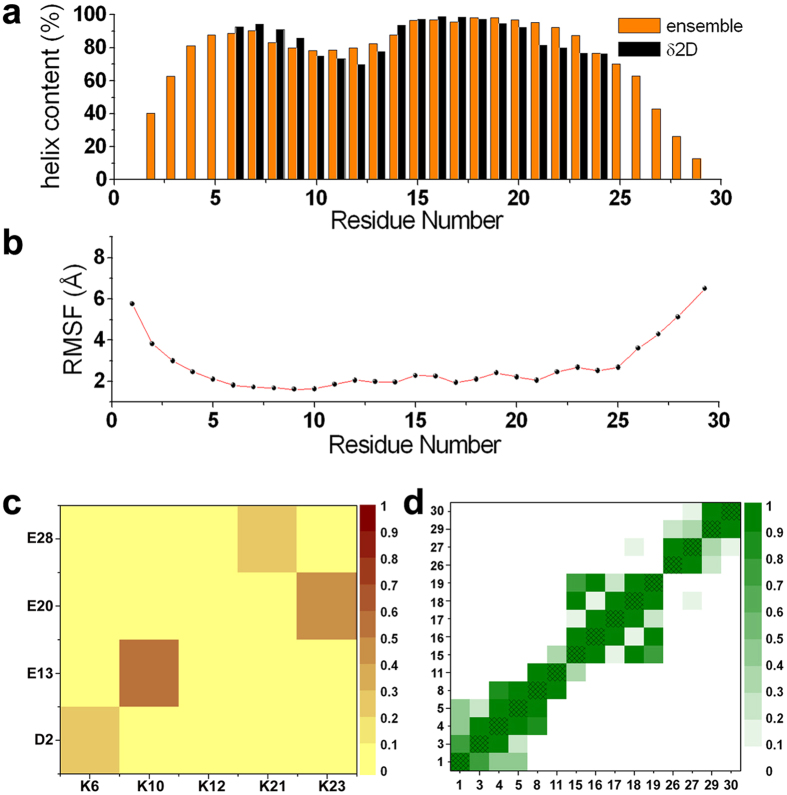
Structural properties of the αS_1-30_ ensemble. (**a**) Comparison between the population of α-helix along the sequence of αS_1-30_ in the structural ensemble, calculated by using the DSSP^61^ program, and that estimated from the analysis of chemical shifts by means of δ2D^37^, which estimates the populations of secondary structure elements using a statistical mechanics approach to interpret the CS values. (**b**) Root mean square fluctuations (RMSF), reporting the standard deviations of the position of C^α^ atoms in the ensemble. (**c**) Identification of salt bridges in the ensemble, calculated using a cutoff of 5.0 Å between the centres of masses of the charged groups of the sidechains. The occurrence of the salt bridges in the ensemble is color coded from 0 (yellow) to 1 (brown). (**d**) Identification of hydrophobic contacts, calculated using a cutoff of 5.0 Å between the centres of masses of the hydrophobic sidechains. The axes indicate residue numbers. The occurrence of hydrophobic contacts in the ensemble is color coded from 0 (white) to 1 (dark green).

The fact that the ensemble of structures of αS_1-30_ in the membrane-bound state has a significant α-helical content is in agreement with independent estimates of the helical population calculated from an algorithm using statistical mechanics of the CS data (δ2D^37^, Fig. 2a). Moreover, the major fluctuations in the structural ensemble of this segment involve the five N-terminal residues and the region spanning residues 26 to 30 (Fig. 2b), in line with previous findings that the most rigid helical region in the membrane-bound state of this segment of αS spans residues 6 to 25^25^. Indeed, residues 6 to 25 show sufficient rigidity to enable a number of cross-peaks in multidimensional ssNMR cross polarisation (CP) experiments to be observed, allowing assignment of its backbone resonances^25^. In contrast with the present structural ensemble, the solution NMR structures of the micelle-bound state of αS (PDB codes 1qx8 and 2kkw) occupy a region of our FES that is indicative of conformations that are very close to the ideal α-helix (Fig. S3a). The reduced structural variability amongst the 1qx8 and 2kkw structures (Fig. S3b), as compared with the present CS-restrained ensemble, is possibly due to the strong interactions that αS establishes with detergent micelles, which result in very significant stabilisation of the helical conformation (Fig. S3c,d).

The FES defined in the present work indicates the nature of the key interactions that stabilise the optimal conformation of αS for membrane binding. In particular, a fundamental element of conformational stabilisation is a network of salt bridges, with probabilities ranging from 30% to 70% within the conformational ensemble (Fig. 2c). These salt bridges, which are not described in the NMR structures of the micelle-bound state of αS (PDB codes 1qx8 and 2kkw), include those formed between the pairs of residues K6/D2, K10/E13, K21/E28 and K23/E20, although, that formed between K21 and E28 is not present in the conformations within the major basin of the FES (Fig. S4). Another key element in the stabilisation of the amphipathic helical conformation of αS_1-30_ is revealed in the FES as an extended network of highly populated exposed hydrophobic patches (Figs 2d and S5). Such hydrophobic patches are likely to stabilise the helical structure in the presence of extended hydrophobic surfaces such as those of lipid bilayers^19^,^20^,^25^,^26^,^27^, detergent assemblies^29^ and water/air interfaces^38^. Indeed, the NMR structures of the micelle-bound state of αS (PDB codes 1qx8 and 2kkw) describe a similar pattern of hydrophobic interactions, although with generally higher occupancy factors than those sampled in the present study (Fig. S3d,e).

### Accurate estimation of the topology of membrane-bound αS

A detailed characterisation of the topological properties of the membrane-anchor region of αS at the surface of SUVs is crucial for elucidating the biological behavior of the protein. In order to characterise this fundamental aspect of the membrane bound state of αS, we performed CS-restrained MD simulations of αS_1-30_ in explicit DOPE:DOPS:DOPC bilayers and explicit waters, using simulation procedures that we have described previously^39^. The simulations, totalling 1 μs in length, were restrained using experimental data measured from full length αS bound to SUVs.

**Figure 3:**
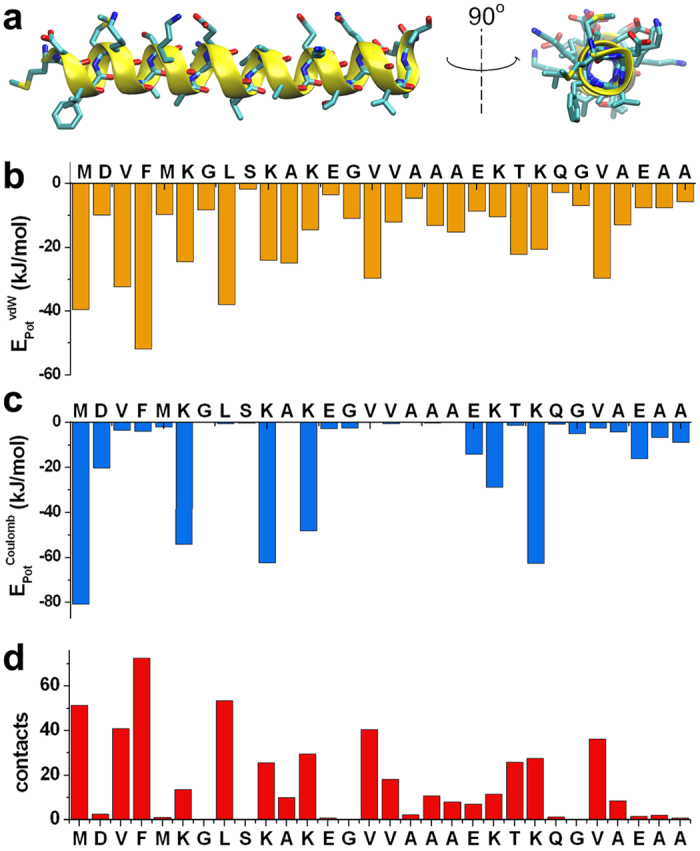
Interaction terms between αS_1-30_ and a DOPE:DOPC:DOPS lipid bilayer. (**a**) Ribbon representation of a representative conformation from the main basin in the FES of αS_1-30_ (Fig. 1). Sidechains are represented by sticks. (**b–d**) van der Waals’ (**b**) and Coulomb (**c**) energies (kJ/mol) for the sidechain-lipid interactions in CS-restrained simulations (see Methods). (**d**) Average number of sidechain-lipid contacts in the ensemble. Contacts are identified using a cutoff of 5.0 Å between the heavy atoms of the sidechains and the lipids.

**Figure 4:**
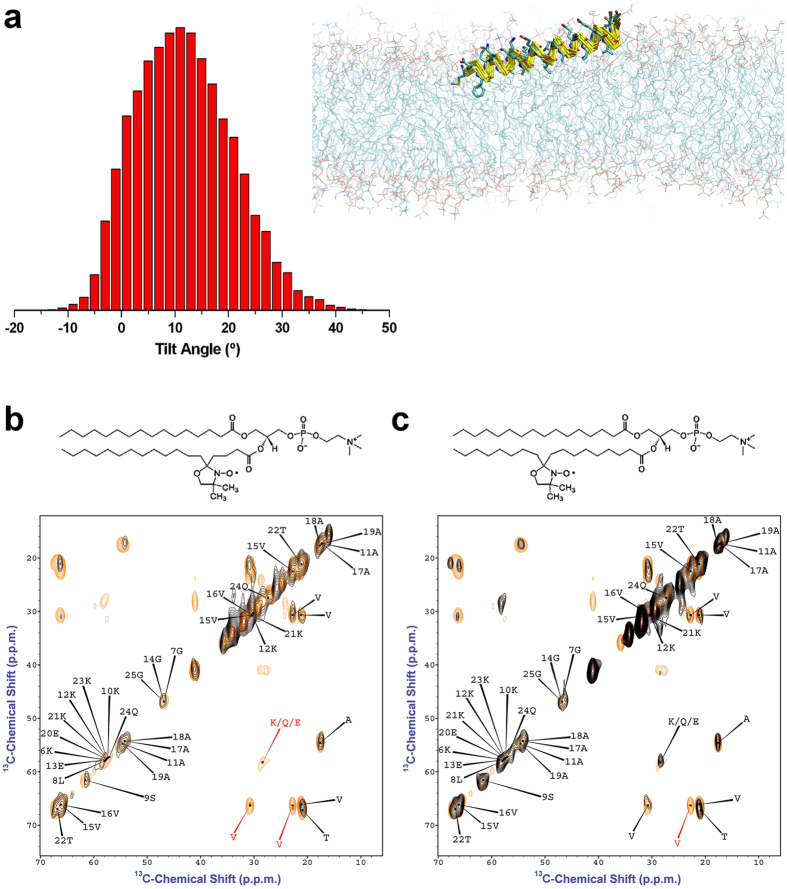
Orientation αS_1-30_ bound to lipid bilayers. (**a**) Orientations of αS_1-30_ with respect to the membrane surface in the structural ensemble generated by using CS-restrained simulations. The tilt angle is that between the axis of the helix and its projection on the surface of the membrane. The latter is interpolated across the phosphorus atoms of the head groups of the lipid molecules that are positioned within 10 Å of any of the protein atoms. (**b**) PRE of full length αS bound to DOPE:DOPS:DOPC SUVs doped with 2% of 1-palmitoyl-2-stearoyl-[5-doxyl]-sn-glycero-3-phosphocholine, which carries an unpaired electron at the position of carbon 5 of the lipid tail. The chemical structure of the paramagnetically labelled lipid is shown at the top of the figure. The lower panel shows the ^13^C-^13^C-DARR spectra of the membrane-bound αS measured as described previously^25^ at mixing and contact times of 50 ms and 1 ms, respectively, and in the presence (black) and absence (orange) of the paramagnetically labelled lipid. Under the conditions employed in this study, the ^13^C-^13^C-DARR spectra can detect the region spanning residues 6 to 25 of the membrane-bound αS. Red labels indicate the pattern of selective peak attenuations, indicating a spatial proximity to the paramagnetic probe. (**c**) PRE experiments performed using 1-palmitoyl-2-stearoyl-[10-doxyl]-sn-glycero-3-phosphocholine, which carries an unpaired electron at position of the carbon 10 of the lipid tail.

The restrained simulations provide a detailed description of the energy of interaction between αS_1-30_ and hydrophilic/hydrophobic regions of the DOPE:DOPS:DOPC bilayers (Fig. 3). In particular, the results indicate the presence of strong stabilising van der Waals’ interactions between the side chains of residues M1, V3, F4, L8 and the hydrophobic groups of the lipid tails (Fig. 3b), which result from a number of intermolecular contacts between these residues and the lipid chains (Fig. 3d). Electrostatic interactions between M1, K6, K10 and K12 and the charged groups of the lipids are also observed, evidencing a stabilising factor for the membrane binding by the N-terminal region of αS_1-30_ (Fig. 3c), which represents a consistent key factor within different membrane binding systems^40^,^41^,^42^. Overall these data indicate that the initial 12 residues of the protein sequence enable αS to establish tight interactions with the membrane, including the internal hydrophobic region of the lipid bilayer. These interactions were found to be associated with a tilt angle (θ) of 12° (Fig. 4a) between αS_1-30_ and the membrane surface suggesting a degree of partial insertion in the membrane.

The observed tilt angle of *ca* 12^o^ with respect to the lipid bilayer of the helical segment of the N-terminal region of αS requires a partial insertion of the N-terminal residues in the hydrophobic region of the membrane. In order to obtain a detailed characterisation of this topological feature, we calculated the average positions of the amino acid residues in the αS_1-30_ simulations with respect to the membrane normal (Fig. S6). This analysis indicated that residues in the region 1–12 are up to 8 Å more deeply inserted into the lipid bilayer than those of the rest of the membrane-anchor region. This is exemplified with F4 and A18, the side chains of which are both located on the same side of the amphipathic helical conformation of αS, which show average positions on the membrane normal of 8.9 Å and 16.9 Å from the centre of the lipid bilayer, respectively (Fig. S6b). This analysis also reveals that the five N-terminal residues of αS, which establish key electrostatic and van der Waals interactions with the membrane (Fig. 3), are associated with a higher degree of variability along the membrane normal than those of residues 6–25. This finding suggests that the local conformational heterogeneity of the region 1 to 5 is responsible for the broadening that prevents the assignment of the resonances of these residues in ^13^C-^13^C-DARR spectra^25^.

In order to gain further experimental evidence for or against the partial insertion of αS into the lipid bilayer^28^, we employed paramagnetic relaxation enhancement (PRE)^43^,^44^ experiments to probe if transient contacts occur between αS and the interior of the lipid bilayer. In our previous magic angle spinning (MAS) study of αS binding to DOPE:DOPS:DOPC SUVs^25^, PRE data were obtained in the presence of paramagnetic labels placed in the hydrophilic head groups, using the gadolinium salt of 1,2-dimyristoyl-sn-glycero-3-phosphoethanolamine-N-diethylenetriaminepentaacetic acid, and at the position of carbon 16 of the lipid tail, using 1-palmitoyl-2-stearoyl-[16-doxyl]-sn-glycero-3-phosphocholine. These two PRE experiments generated marked spectral differences, with only the measurement performed by placing the spin label in the head group of the lipid resulting in selective peak broadening in ^13^C-^13^C-DARR and INEPT spectra. Based on the present restrained simulations, indicating that the first twelve residues of αS can be partially inserted into the lipid bilayer upon binding, we carried out PRE experiments that could probe the degree of insertion of this region by using spin labelled lipids with unpaired electrons at the positions of carbons 5 and 10 of the lipid tail (Fig. 4b,c). In the first case, the ^13^C-^13^C-DARR spectra (Fig. 4b) show the selective broadening of cross peaks corresponding to the side chains of valine (C^α^-C^β^ and C^α^-C^δ^ cross correlations) and lysine residues (C^α^-C^β^ cross correlations). This result suggests that these groups of the protein are spatially close to the unpaired electron at the position of carbon 5 of the lipid tail. The paramagnetic broadening of the resonances in the ^13^C-^13^C-DARR, however, is almost completely absent when the PRE experiments are performed by using spin labelled lipids with unpaired electrons at the position of carbon 10 of the lipid tail (Fig. 4c), which defines a mild level of insertion of αS into the lipid bilayer.

## Discussion

In this study, we have characterised the nature of the protein-lipid interactions that the N-terminal region of αS, which acts to anchor the protein to membranes^25^, establishes with the surface of synaptic-like lipid vesicles. The finding that resonances of this segment of αS are detectable in cross polarisation spectra measured at the magic angle indicates that this region of αS associates with the lipid bilayer with significant affinity, despite being in rapid equilibrium between its bound and unbound states (with a conversion rate of *ca* 200 ms^20^).

In order to define in detail the characteristics of this lipid-binding region, we generated an atomic resolution structural ensemble of the N-terminal 30 residues of αS region bound to DOPE:DOPS:DOPC SUVs by using MD simulations restrained with experimental chemical shifts. The resulting FES reveals the structural determinants of the interaction of this region of αS with membrane bilayers. In addition, we have probed the local topology of this region of the protein with respect to the lipid bilayer by using both restrained MD simulations and ssNMR PRE experiments. These studies indicate that αS_1-30_ adopts a topology in its membrane bound state that involves a partial insertion of the initial 12 residues into the region occupied by hydrophobic chains of the lipid bilayer. The strong intermolecular interactions between the lipid bilayer and the N-terminal 12 residues of αS, as described in the present study, indicate that this region plays a key role in anchoring αS on the membrane surface, which is in line with literature results showing that the deletion of the segment 2-11 causes dramatic impairment of vesicle binding *in vitro* as well as of membrane-binding and cellular toxicity in yeast^45^. The partial membrane insertion of the initial 12 residues endows αS with the ability to tightly bind lipid vesicles while maintaining a rapid equilibrium between membrane bound and unbound states. This equilibrium is thought to be strongly linked to the ability of αS to facilitate the interactions between lipid vesicles that lead to the fusion of synaptic-like SUVs *in vitro*^20^ and to the clustering of synaptic vesicles *in vivo*^18^,^46^,^47^. The ability to promote such interactions has been associated with the putative role of αS in the regulation of the homeostasis of synaptic vesicles at their axon terminal^17^,^48^, by contributing to the maintenance of the optimal pool of synaptic vesicles prior to neurotransmitter release^15^,^16^,^49^. Thus the topological and structural properties identified in the present study for the membrane-bound state of αS provide an explanation of one of the key factors enabling αS to exert its role in the regulation of synaptic vesicles.

Under some circumstances, however, the tight binding of the N-terminal anchor to the surface of lipid membranes can have detrimental effects by favouring the population of conformational states in which the non amyloid-β component (NAC) region of αS, which has been associated with αS aggregation^4^,^50^,^51^, is exposed to the solvent by partial detachment from the SUV surface. As a result, the partial insertion of residues 1–12 of αS into lipid bilayers is potentially not only a key aspect of its functional state but is also the initial step for the aggregation of αS at the surface of lipid vesicles^23^, a process that is associated with the onset and the progression of Parkinson’s disease. Indeed, rather than actively contributing to the fibrillar core of the amyloid state of αS, the current data suggest that the N-terminal region may play a role during the aggregation at the surface of lipid vesicles as a result of its anchoring role to the membrane surface, which in turn enables the amyloidogenic NAC region to exist in equilibrium between membrane-bound and membrane-detached states^25^. The partial detachment of the NAC region from the membrane surface and the general reduction of the degrees of freedom of αS in the membrane-bound state are favourable conditions to promote αS fibrillisation via primary nucleation^23^. This is model is in line with a recent ssNMR structural refinement of the αS fibrils showing that the N-terminal region is not part of the amyloid core^52^.

In conclusion, the present data indicate that the characterisation of the molecular and structural basis of the partition between membrane bound and free states of αS is essential to identify the underlying mechanisms of αS function as well as the nature of the factors that lead to its central involvement in neurodegenerative disorders.

## Methods

### Sample purification

αS was purified in *E. coli* using the plasmid pT7-7 encoding for the protein as previously described^25^. A brief description of the protocol is provided in the Supplementary Materials.

### Paramagnetic Relaxation Enhancement

PRE data were measured with magic angle spinning using a 14.09T Bruker Ascend magnet with Avance *III* HD console and equipped with a 3.2 mm E^Free^ probe (Bruker, Billerica, USA). Dipolar assisted rotational resonance (DARR) experiments^53^ were performed at a MAS rate of 10.0 kHz at −19 °C and 4 °C (the latter for control experiments only), using a 1 ms contact time and 20 ms mixing time in ^13^C-^13^C cross polarization experiments. PRE data were measured by using unlabelled membranes to measure a reference spectrum and then obtaining spectra by using paramagnetic labeled membranes doped with 2% 1-palmitoyl-2-stearoyl-[5-doxyl]-sn-glycero-3-phosphocholine or 2% 1-palmitoyl-2-stearoyl-[10-doxyl]-sn-glycero-3-phosphocholine (Avanti Polar Inc., Alabaster, USA), which carry an unpaired electron in their doxyl group positioned at the level of the 5^th^ and 10^th^ carbon atom of the lipid chain, respectively (Fig. 4b,c).

### Structural ensemble refinement using chemical shift restrained MD simulations

Chemical shifts were employed in restrained molecular dynamics simulations using the CamShift method^33^ to refine accurate structural ensembles of the anchor region of αS. These methods have been largely described in literature. A brief description is provided in the Supplementary Materials.

The restrained molecular dynamics simulations were performed by averaging chemical shift restraints over four replicas, as previously described^35^. The calculations were made using an implementation of the GROMACS package^54^ that allows the simulations to be restrained using the CamShift program^33^. Each of the four replicas was equilibrated separately by starting from random conformations accommodated in a dodecahedron box of 276 nm^3^ in volume. The box was filled with explicit waters and energy minimized. For each replica, the system was thermally equilibrated during a NVT simulation of 250 ps in which the temperature was increased from 10 K to 278 K. Subsequently the pressure was equilibrated at 1 Atm for a 200 ps MD simulation. Finally, the individual replicas were equilibrated for 1 ns long simulations.

The replica-averaged restrained molecular dynamics simulations were carried out using the following protocol. The four replicas evolved through a series of annealing cycles between 278 K and 350 K, each cycle being composed of 100 ps of simulation at 278 K followed by 100 ps of simulation in which the temperature of the system was increased up to 350 K and 100 ps of simulation carried at a constant temperature of 350 K. The final part of the cycle allowed the system to cool slowly from 350 K to 278 K in a step of 300 ps. During these cycles the experimental restraints were imposed as averages over the four replicas according to equations 1–3 (Supplementary Information). The total amount of sampling in each system simulated was 1 s (250.2 ns *per* replica equivalent to 417 cycles). The first refinement of the structural ensemble of the anchor region of αS was performed by starting from four random conformations generated by a single 100 ns all atom simulation (NPT ensemble run at 500 K) starting from a linearised protein with the sequence of αS_1-30_. The second ensemble, involving the interaction with the membrane, was started by equilibrating the representative minimum in the FES generated with the first ensemble in the presence of a pre-equilibrated membrane bilayer composed of DOPE:DOPS:DOPC lipids in a ratio 5:3:2.

The simulations were carried out using the AMBER99sb-ILDN force field^55^ and the Tip4pEW^56^ water model. In the case of simulations performed in the presence of the lipid bilayer, we employed the parameters of the all-atom force field defined for phosphatidylcholine lipids^57^. The protonation states of pH-sensitive residues were as follows: Arg and Lys were positively charged, Asp and Glu were negatively charged, and His had zero charge and the net charge of the system was neutralized by the addition of Na^+^ and Cl^−^ ions. A time step of 2 fs was used together with LINCS constraints^58^. The van der Waals and electrostatic interactions were cut off at 0.9 nm, and long-range electrostatic effects were treated with the particle mesh Ewald method^59^. All the simulations were carried out in the canonical ensemble by keeping the volume fixed and by setting the system temperature with the V-rescale thermostat^60^. The final samplings were collected from the 278 K portions of the replica simulations after discarding the initial 50 ns in each replica, which represented the equilibration phases of the ensemble. The total number of conformations collected in each sampling was 13320 and convergence was tested using four different structural parameters (Fig. S1).

## Additional Information

**How to cite this article**: Fusco, G. *et al*. Structural Ensembles of Membrane-bound α-Synuclein Reveal the Molecular Determinants of Synaptic Vesicle Affinity. *Sci. Rep*. **6**, 27125; doi: 10.1038/srep27125 (2016).

## Supplementary Material

Supplementary Information
