# Supplementary material for: Structural Ensembles of Membrane-bound α-Synuclein Reveal the Molecular Determinants of Synaptic Vesicle Affinity
Source: Sci Rep. 2016 Jun 8;6:27125. doi: 10.1038/srep27125 (PMC4897633; doi:10.1038/srep27125)
Supplement: Supplementary Information [file srep27125-s1.pdf]

Supplementary Information

Structural Ensembles of the Membrane-bound α-Synuclein Reveal the Molecular Determinants for Synaptic Vesicle Affinity

Giuliana Fusco[1], Alfonso De Simone[2], Paolo Arosio[1], Michele Vendruscolo[1], Gianluigi Veglia[3], Christopher M. Dobson[1]

[1]*Department of Chemistry, University of Cambridge, Lensfield Road, Cambridge, CB2 1EW, UK.*
[2]*Department of Life Sciences, Imperial College London, South Kensington, London, SW7 2AZ, UK.*
[3]*Department of Chemistry & Department of Biochemistry, Molecular Biology & Biophysics, University of Minnesota, 6-155 Jackson Hall 321 Church st. SE, Minneapolis, MN 55455, USA*

Supplementary Materials and Methods

αS purification.

After transforming in BL21 (DE3)-gold (Agilent Technologies, Santa Clara, CA) cells, uniformly ^{15}N and/or ^{13}C labeled αS was obtained by growing the bacteria in isotope-enriched M9 minimal media containing 1g/L of ^{15}N-NH_4Cl and 2g/L of ^{13}C-glucose (Sigma-Aldrich, St Louis, USA). The cells were grown at 37 °C under constant shaking at 250 rpm in LB medium, supplemented with 100 μg/ml ampicillin, to an OD600 of 0.6. Subsequently the expression of the protein was induced with 1mM IPTG at 37 °C for 4 h, and the cells were harvested by centrifugation at 5,000 rpm (Beckman Coulter, Brea, USA). The cell pellet was resuspended in lysis buffer (10mM Tris-HCl pH 8, 1mM EDTA and EDTA-free complete protease inhibitor cocktail tablets - Roche, Basel, Switzerland) and lysed by sonication. The cell lysate was centrifuged at 13,500 rpm for 30 min to remove cell debris. The supernatant was then heated for 20 min at 70°C and centrifuged at 13,500 rpm in order to precipitate heat-sensitive proteins. Subsequently streptomycin sulfate was added to the supernatant to a final concentration of 10 mg/ml to stimulate DNA precipitation. The mixture was stirred for 15 min at 4°C followed by centrifugation at 13,500 rpm. Then, ammonium sulfate was added to the supernatant to a concentration of 360 mg/ml in order to precipitate the protein. The solution was stirred for 30 min at 4°C and centrifuged again at 13,500 rpm. The resulting

pellet was resuspended in 25 mM Tris-HCl, pH 7.7 and dialyzed against the same buffer in order to remove salts. Dialyzed solutions were loaded onto an anion exchange column 26/10 Q sepharose high performance (GE Healthcare, Little Chalfont UK) and eluted with a 0–1 M NaCl step gradient, and then further purified by loading onto a size exclusion Column Hiload 26/60 Superdex 75 preparation grade (GE Healthcare, Little Chalfont UK). All the fractions containing the monomeric protein were pooled together and concentrated by using Vivaspin filter devices (Sartorius Stedim Biotech Gottingen, Germany). The purity of the aliquots after each step was analyzed by SDS-PAGE and the protein concentration was determined from the absorbance at 275 nm using an extinction coefficient of 5600 M^{-1} cm^{-1}.

Implementation of the chemical shift restraints in MD simulations.

CS restrained simulations were run with a modified version of the GROMACS package[1] that allows restraining the simulations using the CamShift program[2]. Briefly, the restraints were imposed by adding a pseudo-energy term (E^{CS}) to a standard molecular mechanics force field (E^{FF}):

$$E^{Tot} = E^{FF} + E^{CS} \quad (1)$$

The resulting force field (E^{Tot}) was employed in molecular dynamics simulations, where the pseudo-energy term is given by

$$E^{CS} = \alpha \sum_i (\delta_i^{Exp} - \delta_i^{Calc})^2 \quad (2)$$

where the *i* sum runs over all the chemical shifts employed in the refinement, α is the weight of the restraint term, and δ^{exp} and δ^{calc} are the experimental and calculated chemical shifts, respectively. We employed the replica-averaged scheme which implies that a given chemical shift is calculated by

$$\delta^{Calc} = \frac{1}{m} \sum_m^4 \delta_m^{Calc} \quad (3)$$

where m runs over four replicas and δ_m^{Calc} is the chemical shift of replica m.

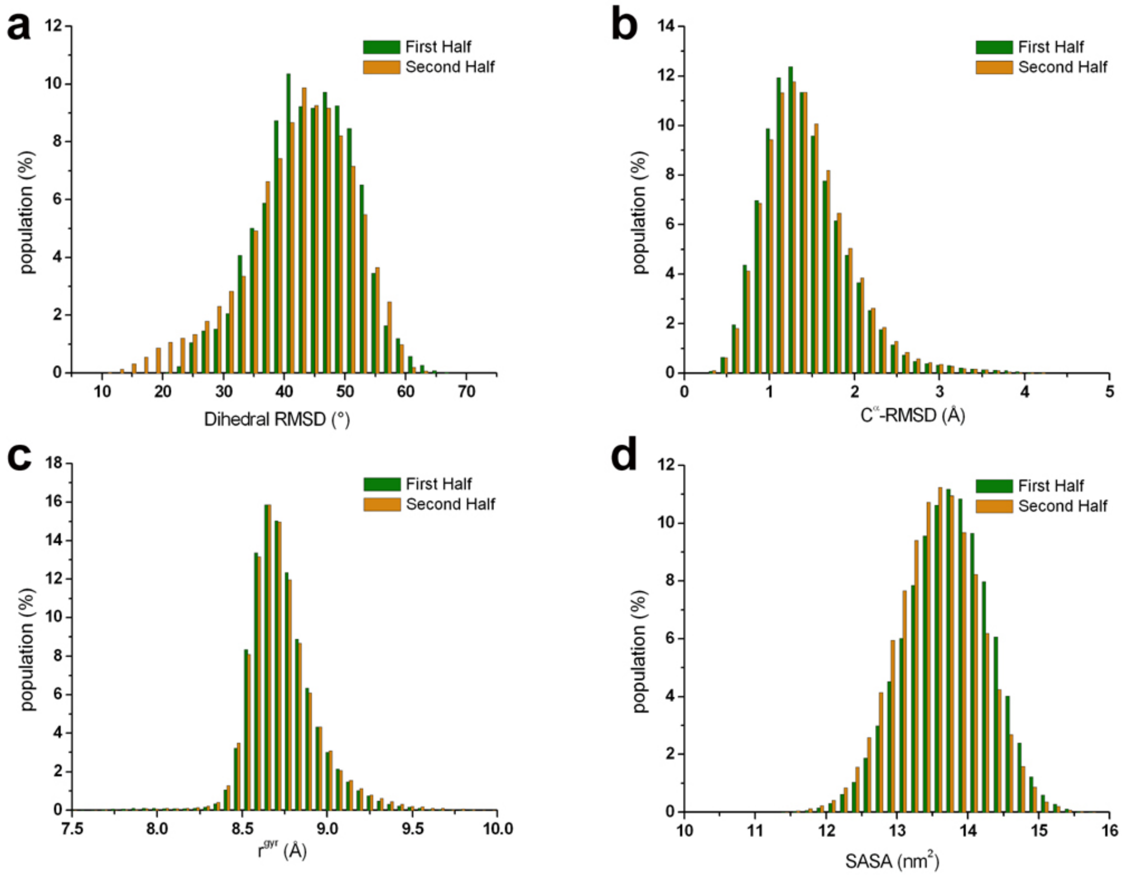

Supplementary Figure S1. Convergence of the chemical shift restrained simulations of αS$_{1\text{-}30}$. To check the convergence of the simulations, the resulting ensemble was divided in two parts, with the first half (green histograms) being composed of the 50 ns to 150 ns segment of each of the four replicas of the sampling, and the second half (orange histograms) being composed of the 150 ns to 250 ns segment. The segment 0 ns to 50 ns was disclosed from this analysis as it represents the equilibration phase. Comparison of the distributions of the values of dihedral RMSDs (phi and psi angles), C$^{\alpha}$-RMSDs, radii of gyration and surface accessibility areas are shown in panels a, b, c, and d, respectively.

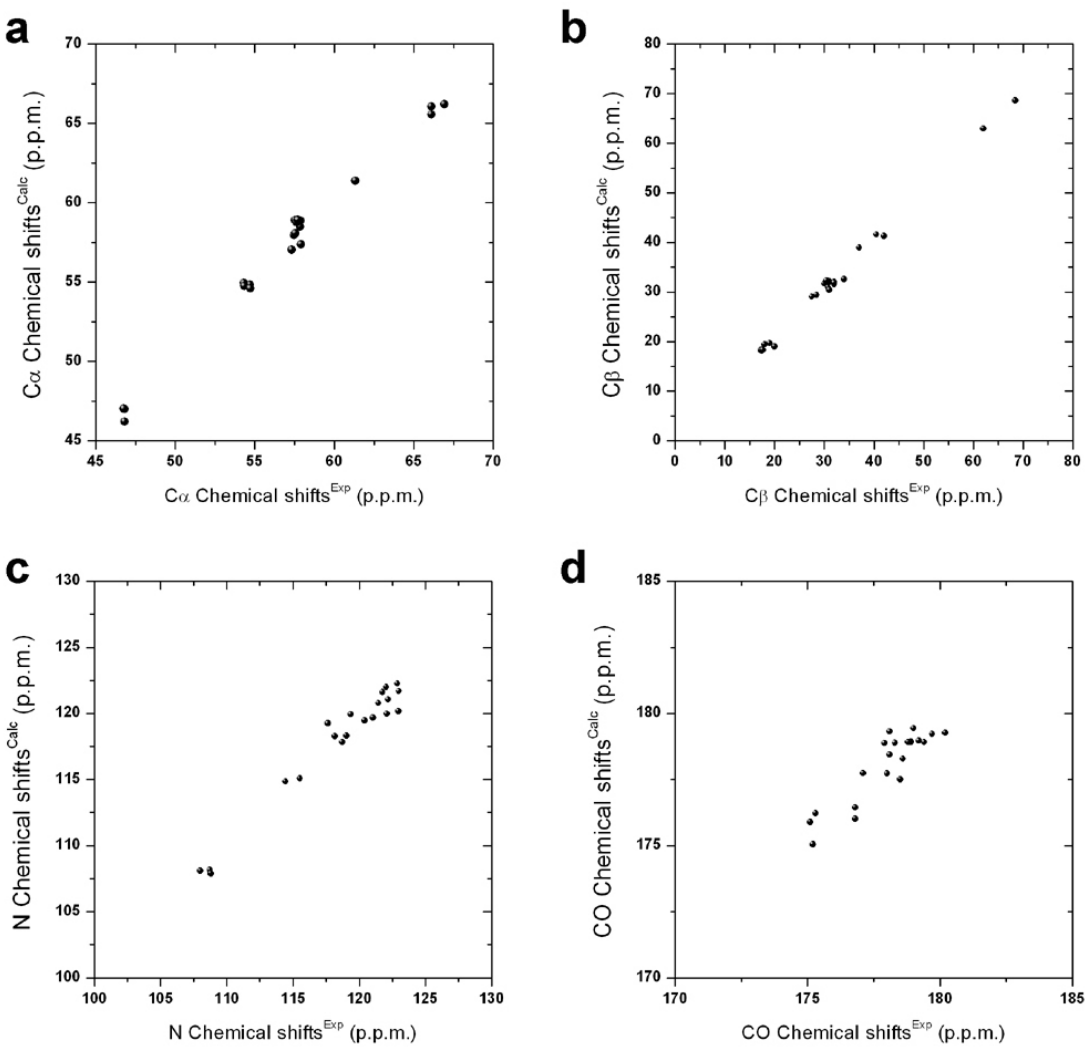

Supplementary Figure S2. Validation of the CS-restrained simulations of $\alpha S_{1\text{-}30}$. Validation of the structural ensemble of $\alpha S_{1\text{-}30}$ was made by comparing the experimental chemical shifts and those back calculated from the structures by using the SPARTA+[3]. As SPARTA+[3] is based on principles than are different from those of CamShift[2], as employed in the present study to restrain the MD simulations, the high level of agreement provides an independent validation of the quality of the ensembles. The standard deviations are within the statistical errors of SPARTA+ for all the atoms, giving an indication of the high quality of the structural ensembles. Validation for resonances corresponding to Cα, Cβ, N, and CO atoms is shown in panels a, b, c and d, respectively.

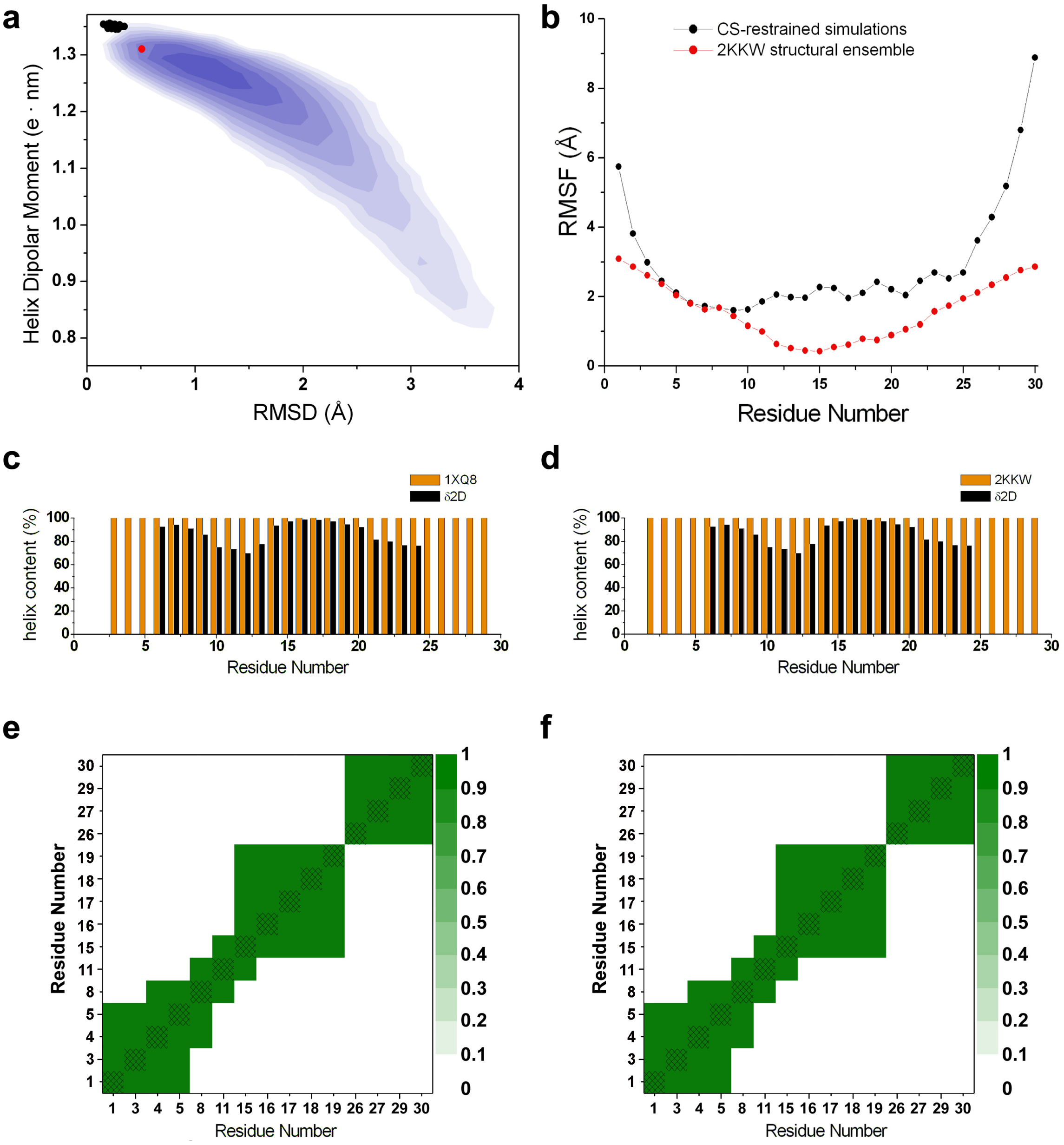

Supplementary Figure S3. Structural features of micelle-bound αS structures. a) Projections of the NMR structures of the micelle-bound state of αS (PDB codes: 1xq8 and 2kkw for red and black dots, respectively) on the FES generated in this study (Fig. 1). **b)** Root mean square fluctuations (RMSF), reporting the standard deviations of the position of C^{α} atoms in the CS-restrained ensemble (black) and the PDB structure 2kkw (red). **c-d)** Comparison between the population of α-helix along the sequence of $\alpha S_{1\text{-}30}$ the PDB structures 1xq8 and 2kkw (region 1-30 only), calculated by using the DSSP program[59] (orange), and that estimated from the analysis of chemical shifts by means of δ2D[37] (black), which estimates the populations of secondary structure elements using a statistical

mechanics approach to interpret the CS values. **b) e-f.** Map of the contacts of the hydrophobic patches in the PDB structures 1xq8 and 2kkw (region 1-30 only). Hydrophobic contacts are identified using a cutoff of 5.0 Å between the centres of masses of the hydrophobic sidechains.

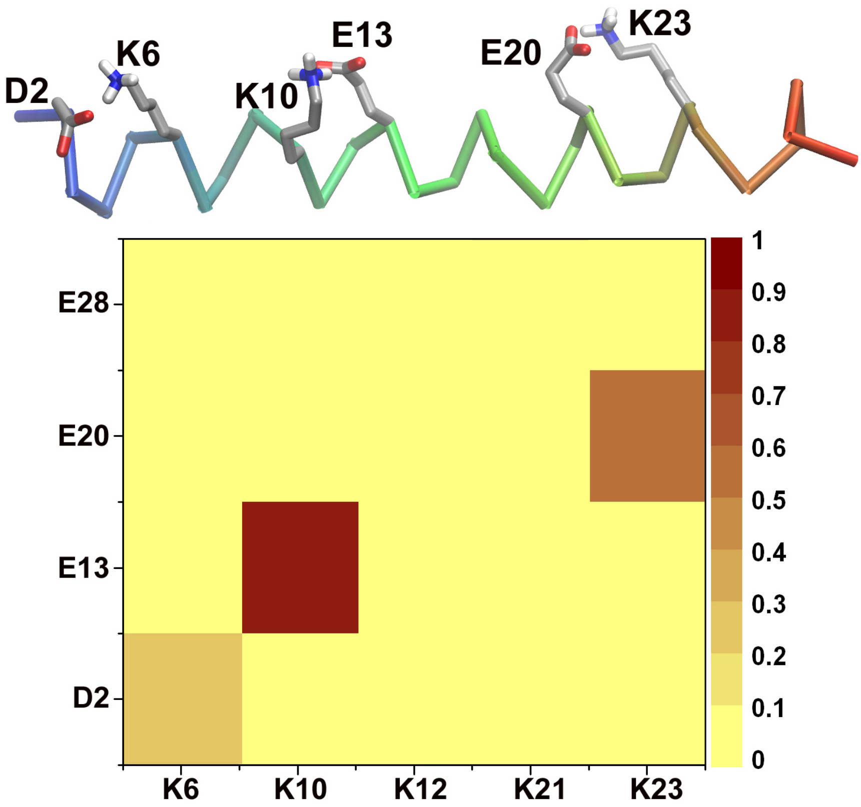

Supplementary Figure S4. Map of the occurrence of salt bridges in the main basin of the FES of αS$_{1\text{-}30}$ (Fig. 1). Salt bridges are identified using a cutoff of 5.0 Å between the centres of masses of the charged groups of the sidechains. The populations of each salt bridges in the ensemble are color coded from 0 (yellow) to 1 (brown). The structure above is the representative conformation of the main basin of the FES (Fig. 1).

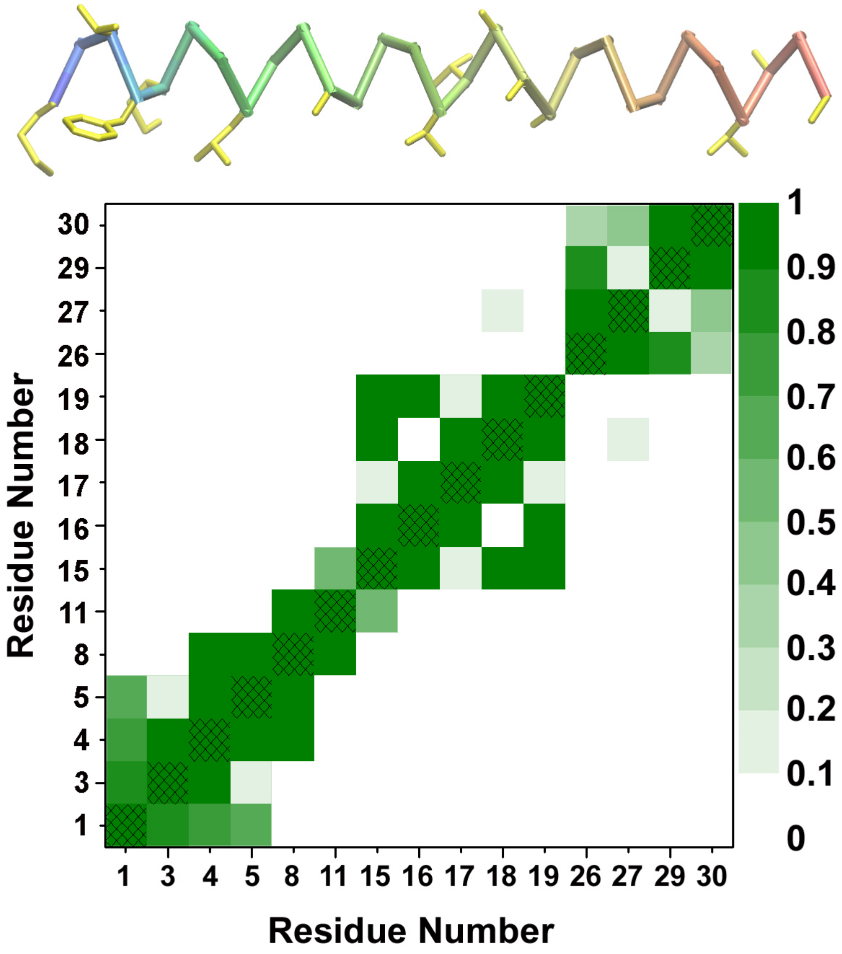

Supplementary Figure S5. Map of the contacts of the hydrophobic patches in the main basin of the FES of αS$_{1\text{-}30}$ (Fig. 1). Hydrophobic contacts are identified using a cutoff of 5.0 Å between the centres of masses of the hydrophobic sidechains. The populations of each hydrophobic contact in the ensemble are color coded from 0 (white) to 1 (green). The structure above is the representative conformation of the main basin of the FES (Fig. 1).

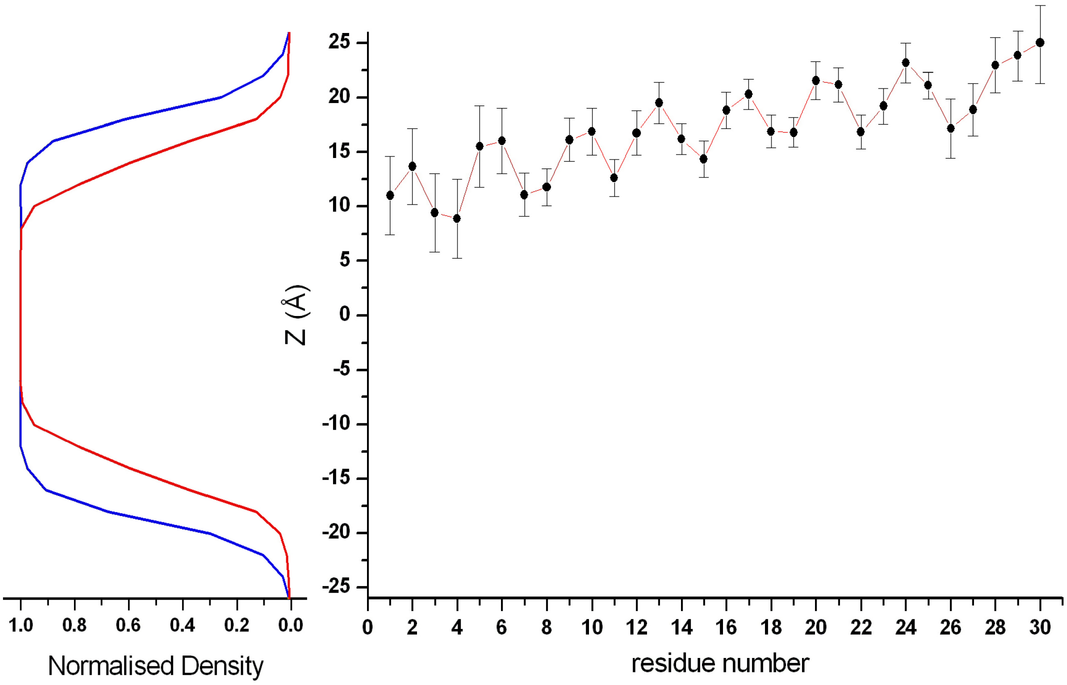

Supplementary Figure S6. Positions of residues of αS$_{1\text{-}30}$ on the membrane normal. a) Normalised density of atoms from the lipid molecules in the direction of the membrane normal (blue). Red curve includes atoms from hydrophobic groups of the lipids only. **b)** Average positions of the centre of mass of each residue of the αS$_{1\text{-}30}$ projected on the membrane normal. The plot is referenced in the frame of the membrane, such that the centre of the lipid bilayer assumes a value of 0 on the membrane normal.

References

1. Hess B, Kutzner C, van der Spoel D, Lindahl E. GROMACS 4: Algorithms for highly efficient, load-balanced, and scalable molecular simulation. *Journal of chemical theory and computation* **4**, 435-447 (2008).

2. Kohlhoff KJ, Robustelli P, Cavalli A, Salvatella X, Vendruscolo M. Fast and accurate predictions of protein NMR chemical shifts from interatomic distances. *Journal of the American Chemical Society* **131**, 13894-13895 (2009).

3. Shen Y, Bax A. SPARTA+: a modest improvement in empirical NMR chemical shift prediction by means of an artificial neural network. *Journal of biomolecular NMR* **48**, 13-22 (2010).
